# An Emerging Way for Bacteria to Engage with Host Cells via Protein ADP-riboxanation

**DOI:** 10.3390/toxins16110467

**Published:** 2024-11-01

**Authors:** Wei Xian, Zhiheng Tang, Qinxin Zhang, Ying Wang, Xiaoyun Liu

**Affiliations:** Department of Microbiology and Infectious Disease Center, NHC Key Laboratory of Medical Immunology, School of Basic Medical Sciences, Peking University Health Science Center, Beijing 100191, China; xianwei@pku.edu.cn (W.X.); zhihengt@pku.edu.cn (Z.T.); 1610305103@pku.edu.cn (Q.Z.); 2011110019@bjmu.edu.cn (Y.W.)

**Keywords:** ADP-riboxanation, ADP-ribosylation, post-translational modifications, bacterial effectors

## Abstract

Post-translational modifications (PTMs) are increasingly recognized as important strategies used by bacterial pathogens to modulate host cellular functions. Protein ADP-riboxanation, a derivative of ADP-ribosylation, has recently emerged as a new biochemical way by which bacterial pathogens interact with host cells. Recent studies have revealed that this modification has broad regulatory roles in host processes including cell death, protein translation, and stress granule formation. Given that the vast majority of bacterial ADP-riboxanases are still uncharacterized, in this review we also highlight the utility of advanced proteomic tools in the functional dissection of ADP-riboxanation events during bacterial infections.

## 1. Introduction

Post-translational modifications (PTMs) allow the functional diversification of proteins and thus play a crucial role in regulating various cellular processes. PTMs can alter the physical and chemical properties of proteins, their conformations, and their interactions with other molecules, thereby exerting an additional layer of regulation on their activity, localization, stability, and overall function. Recently, growing evidence has suggested that bacterial pathogens have evolved sophisticated mechanisms to exploit PTMs and establish successful infections in their mammalian host cells. Of note, many Gram-negative bacterial pathogens are able to deliver effector proteins directly into host cells via specialized bacterial secretion systems. Such bacterial effectors often harbor enzymatic activities that allow them to target and covalently modify key host proteins. We have compiled a list of representative effector-mediated modifications of host proteins (Table 1), which include protein phosphorylation, methylation, glycosylation, deamidation, AMPylation, ADP-ribosylation, and classical and non-canonical ubiquitination and deubiquitination. These PTMs target a wide variety of cellular proteins and host processes, such as immune responses, cell death, vesicle trafficking, autophagy, the cytoskeleton, ubiquitin, and phosphosignaling. Importantly, given the rapidly evolving nature of this field over the last decade, what we show here is only a partial list of the PTMs catalyzed by bacterial effectors. Indeed, these bacterial strategies for post-translationally regulating host cellular processes have been extensively reviewed elsewhere [1,2,3,4,5] and will not be the focus of our current review.

Recently, a new modification known as protein ADP-riboxanation has been characterized in *Shigella flexneri* (*S. flexneri*), which is a common bacterial cause of watery diarrhea and bacillary dysentery worldwide [33,34]. Distinct from ADP-ribosylation, ADP-riboxanation couples the classical attachment of an ADP-ribose moiety with an additional deamination step (i.e., the loss of ammonia). Since its discovery, this new modification has attracted considerable attention in host–pathogen interactions. Understanding these subtle yet significant differences (from classical ADP-ribosylation) is crucial for unraveling the complex mechanisms of bacterial pathogenesis and host defenses. This review will highlight our current understanding of ADP-riboxanation and its roles in host–bacteria interactions.

## 2. Protein ADP-ribosylation and ADP-riboxanation

**Figure 1:**
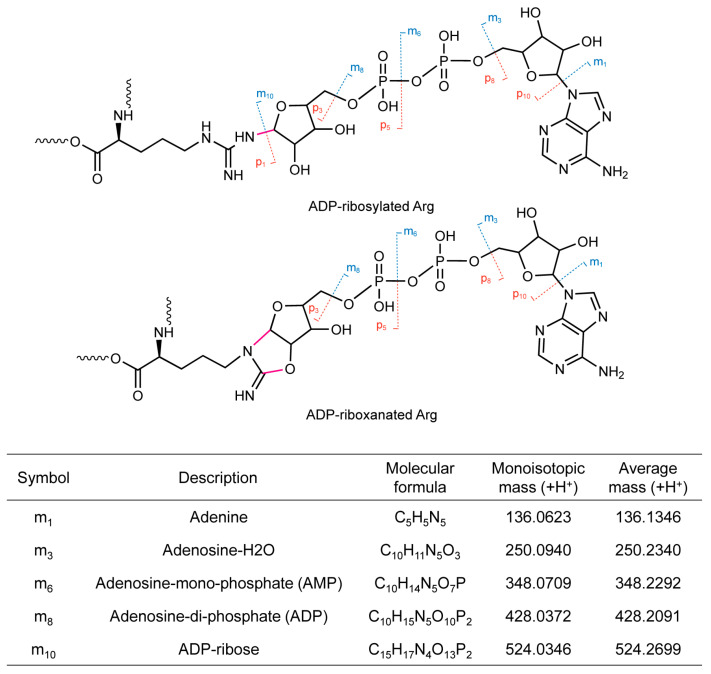
Chemical structures of ADP-ribosylated and ADP-riboxanated arginines. ADP-ribose fragment ions with strong signals in collision-induced dissociation (CID) MS/MS analyses are shown (m1, m3, m6, m8, and m10).

Over the last several years, there has been a renewed surge of attention in reversible adenosine diphosphate (ADP)-ribosylation in host–pathogen interactions. ADP-ribosyltransferases (ARTs) catalyze this modification by transferring one or more ADP-ribose moieties from a nicotinamide adenine dinucleotide (NAD^+^) to a target substrate with the release of nicotinamide (Nam), whereas ADP-ribosylhydrolases (ARHs) remove ADP-ribose from a substrate. The first reported ARTs were cholera and diphtheria toxins from bacteria [35,36]. Based on their conserved structural features in the active center, subsequently identified ARTs have generally been classified into two major groups: cholera toxin-like ARTs (ARTCs) and diphtheria toxin-like ARTs (ARTDs, also known as PARPs) [36]. Recently, we identified an unprecedented PTM dubbed ADP-riboxanation by high-resolution and multi-stage mass spectrometry. This modification is a close derivative of canonical ADP-ribosylation and is catalyzed by the *S. flexneri* type III effectors OspC1/2/3. In ADP-riboxanated proteins, the arginine N^δ^ is the atom where the initial ADP-ribosylation occurs, and subsequently the arginine N^ω^ undergoes additional deamination (Figure 1).

## 3. OspCs

**Figure 2:**
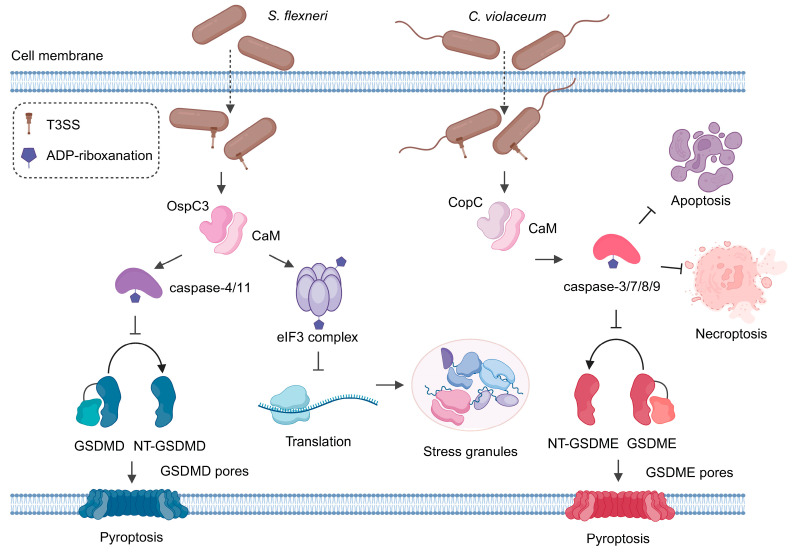
A schematic representation of host–bacteria interactions mediated by ADP-riboxanation. The *S. flexneri* effector OspC3 targets caspase-4/11 to block cleavage of GSDMD and activation of host pyroptosis. NT-GSDMD/GSDME refers to the N-terminal product of GSDMD/GSDME upon caspase cleavage. In addition, the eIF3 complex can be modified by all three OspC effectors, leading to protein translational arrest and stress granule formation. In comparison, the *C. violaceum* effector CopC targets more diverse caspases and inhibits multiple cell death pathways.

The OspC (outer *Shigella* protein C) family comprises the effector proteins OspC1, OspC2, and OspC3, which are secreted by *Shigella* via the type III secretion system (T3SS). Among these proteins, OspC3 has been best characterized due to its prominent role in inhibiting epithelial cell death (i.e., pyroptosis) caused by *Shigella* infection [37]. Recently, OspC3, but not its paralogues OspC1 and 2, was found to covalently modify caspase-4/11 (human caspase-4 and mouse caspase-11). Mass spectrometry analyses together with biochemical dissections uncovered ADP-riboxanation modifications on Arg314 and Arg310 in caspase-4 and caspase-11 [26,38]. Notably, Ca^2+^-free calmodulin (CaM) binds to OspC3 and stimulates its ADP-riboxanase activity. The structures of the CaM–OspC3–caspase-4 ternary complex show that NAD^+^ binding promotes reorganization of the OspC3 catalytic pocket and its engagement with R314/310 of caspase-4/11. In addition, D231 serves as a base to activate the substrate for initial N^δ^-ADP-ribosylation of arginine. Then, D177, as another base, attacks ribosyl 2′-OH in the ADP-ribosylated arginine for subsequent N^ω^-deamination [39]. Functionally, this modification blocks autoprocessing of caspase-4/11 as well as their recognition and cleavage of downstream gasdermin D (GSDMD), thereby preventing GSDMD-dependent anti-*Shigella* humoral immunity and blocking host pyroptosis induced by cytosolic lipopolysaccharides (LPSs) derived from invading bacteria [26] (Figure 2).

Interestingly, OspC1 and OspC2 exhibit significant homology with OspC3 and share similar N-terminal ADP-riboxanation catalytic domains, suggesting highly conserved ADP-riboxanase activity. However, the C-terminal substrate-recognition domains of OspC1/2 diverge from that of OspC3, leading to distinct target specificity. As a result, OspC1/2 do not interact with or modify caspase-4/11 [26]. Of note, Ashida et al. demonstrated that OspC1, but not OspC2 or OspC3, inhibits host apoptosis by preventing caspase-8 activation during *S. flexneri* infection [40]. Therefore, it would be interesting to explore how the specificity of OspC1 targeting apoptosis is established. Additionally, whether such inhibition requires ADP-riboxanase activity remains to be determined. Furthermore, Alphonse et al. reported that OspC1 and OspC3 (but not OspC2) inhibit interferon signaling by binding to CaM and blocking CaM kinase II and downstream JAK/STAT phosphorylation in a process independent of their ADP-riboxanase activity [41]. However, the authors found that all members of the OspC family, including OspC2, have the capacity to interact directly with CaM. This claim seems to be echoed by an independent structural study, which suggests that the CaM binding site has a highly conserved nature among the OspC family [39]. Therefore, it remains undetermined how the specificity of the effectors (i.e., OspC1/3 but not OspC2) is established to dampen interferon responses during *S. flexneri* infection.

## 4. CopC

As a homolog of *S. flexneri* OspC3, the *Chromobacterium violaceum* T3SS effector CopC (*Chromobacterium* outer protein C) harbors the same arginine ADP-riboxanase activity as the OspC family. Therefore, it would not be unexpected if they had similar host targets (i.e., caspases). Indeed, almost at the same time, two studies from different groups confirmed this expectation [7,8]. Nonetheless, CopC seems to target a wider spectrum of substrates in terms of caspases. CopC post-translationally modifies caspase-3/-7/-8/-9 via ADP-riboxanation to inhibit diverse cell death pathways, including apoptosis, necroptosis, and pyroptosis (Figure 2). Interestingly, modifications of these caspases occur through a mechanism that also requires CaM as a cofactor. The stable interaction of CopC-CaM seems to be consistent with the binding of OspC effectors to CaM reported by Alphonse et al. [41]. Furthermore, structural insights into the CaM–CopC–caspase-3 ternary complex reveal the molecular basis of the catalytic and substrate/cofactor binding mechanism. Notably, CopC D172 serves to immobilize R207 of caspase-3 during the modification reaction via a hydrogen bond, while D230 activates a ribosyl 2′-OH of ADPR for additional deamination of ADP-ribosylated arginine. Therefore, mutations of D230 or D172 in CopC abolished caspase modifications both in vitro and during *C. violaceum* infection [42]. Combined with the structural study of the CaM–OspC3–caspase-4 ternary complex, the consensus seems to be that both OspC and CopC effectors require CaM to stimulate their ADP-riboxanase activities. Indeed, growing evidence suggests that dependence on a host cofactor for activation may be a common theme for bacterial effectors delivered into host cells [18,19,43]. In principle, such a necessity would prevent potentially unwanted damage to the bacterial cells where these effectors (toxins-to-be) are initially produced. For example, two recent studies simultaneously reported that the *Legionella* type IV effector LnaB relies on binding to host actin to exert its function as a phosphoryl-AMPylase to activate NF-κB signaling and impair host phosphosignaling pathways in general [18,19].

## 5. Broader Functions of ADP-riboxanation Revealed by Unbiased Proteomics

**Figure 3:**
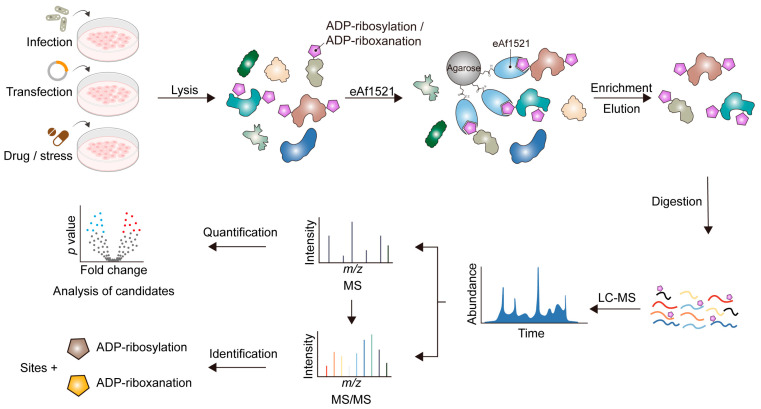
A flow chart that depicts the proteomic approach to global analyses of protein ADP-riboxanation/ADP-ribosylation in mammalian cells.

Thus far, known host substrates of bacteria-mediated protein ADP-riboxanation are still rather limited (i.e., to caspases). However, immunoblotting analyses of mammalian cells expressing OspCs using an antibody specific for ADP-ribose revealed many intense bands spanning a broad range of molecular weights, indicating potentially diverse ADP-riboxanated host targets. Therefore, to gain a holistic view of the host processes regulated by protein ADP-riboxanation, an unbiased systems-level approach would be highly desired to catalogue cellular substrates modified by bacterial effectors during infection. To achieve this goal, we adopted a proteomic strategy initially developed by the Hottiger group [44] to globally map canonical ADP-ribosylation events in mammalian cells (Figure 3). The essence of such a strategy is the utilization of an engineered Af1521 (eAf1521) macro-domain protein with high affinity towards the ADP-ribose moiety. Therefore, ADP-ribosylated proteins can be enriched with high specificity from crude cell lysates prior to identification by mass spectrometry. Given the subtle difference between ADP-riboxanation and the canonical modification, we found that this approach can be applied for comprehensive identification of ADP-riboxanated proteins as well [27].

Notably, we successfully identified the eukaryotic translation initiation factor 3 (eIF3) complex as a novel host target of OspC family effectors. OspCs catalyze modifications of several peripheral subunits of this mega protein complex, including eIF3g, eIF3d, and eIF3j. Given the established role of eIF3 in protein translation, we demonstrated that OspC-catalyzed ADP-riboxanation of eIF3 leads to potent inhibition of global protein synthesis. In fact, it has been reported that several *Legionella* effectors such as Lgt1-3 [45,46] and SidI [47] interfere with host protein translation. Nevertheless, OspCs target translation initiation (rather than elongation), which is usually the rate-limiting step in protein production. In light of this unique feature of OspCs, intriguingly, we further demonstrated that ADP-riboxanation-mediated translation arrest triggers the formation of stress granules in host cells, thereby promoting host survival and pathogen proliferation [27] (Figure 2). Nevertheless, the detailed mechanisms of how stress granules contribute to *S. flexneri* infection remain to be determined. It would be intriguing to explore how the protein composition of *S. flexneri*-induced granules differs from those of classical ones. Understanding their unique composition may shed some light on how stress granules promote *S. flexneri* replication in host cells. In addition, our proteomic dataset indicates the presence of other modified candidates. Currently, we are undertaking efforts to validate other potential targets and follow up with functional studies.

## 6. Limitation(s) of Proteomic Profiling During Target Identification

Despite the clear advantages of ADP-ribosylome profiling during target discovery, such proteomics strategies are not without limitation(s). Before we carried out these experiments, we thought that the known OspCs’ substrates, caspase-4/11, would serve as positive controls in proteomic experiments that were designed to identify potentially ADP-riboxanated host proteins (i.e., enzymatic targets of OspCs). In the end, we did pick up minimal signals derived from a few caspases in cells expressing wild-type OspC1 but not its catalytic mutant. However, due to their relatively low signals in the mass spectrometry analyses, these caspases were not considered to have outstanding differences (or to be top hits) in the large dataset. As a result, they are less likely to be prioritized for further validation and functional studies (if we faithfully follow what the big data tell us).

Having said this, the inability to pick up caspases with ease in the proteomic screen was not unexpected. Despite highly efficient enrichment of ADP-riboxanated substrates, mass spectrometry detection of bona fide targets was largely dictated by their relative abundance in the enriched samples (which also contained endogenously ADP-ribosylated host proteins). Indeed, PARP1 often appeared at the top of the list of identified proteins. Other than abundant background proteins, our top hits (i.e., eIF3 subunits) were likely expressed at much higher levels than caspases in the cells. They appeared as the elephant in the room (which corresponded to the enriched pool of modified proteins). In other words, caspases were simply overshadowed by “the elephant”. Therefore, it is conceivable that our proteomic profiling may have missed some modified substrates that were present at extremely low levels. To overcome such limitations, it would be necessary to carry out these experiments with either extensive fractionation of enriched samples or more sensitive mass spectrometers, both of which would presumably allow us to take a deeper look at the proteome readout.

## 7. Conclusions

The findings on protein ADP-riboxanation have profound implications for our understanding of bacterial pathogen–host interactions. They reveal a novel mechanism through which bacteria, such as *S. flexneri*, can manipulate host cell processes. By modifying key proteins involved in host defense mechanisms, bacterial pathogens can effectively escape host immune responses, thereby promoting infection and pathogen proliferation. Of note, unlike canonical ADP-ribosylation, ADP-riboxanated caspase-4 was resistant to cleavage mediated by ADP-ribosylarginine hydrolase (ADPRH) and other known host ADP-ribosylhydrolases [26]. Some pathogens, especially *Legionella pneumophila*, are known to encode metaeffectors to counteract the activities of effectors [48]. However, bacterial enzymes capable of removing ADP-riboxanation have not been reported thus far. Therefore, OspC-catalyzed modifications are considered, at least for the time being, to be irreversible in host cells.

Functional studies of protein ADP-riboxanation in bacterial virulence are still in their infancy. As previously reported, OspC-like ADP-riboxanases are widely present in diverse bacteria, including *Vibrio*, *Salmonella*, *Erwinia,* and *Chromobacterium* [7,26]. Further elucidation of their host targets and biological functions would be important to enrich our understanding of the roles of this emerging modification in host–pathogen interactions. As we discussed above, new proteomic tools will certainly play an important role in speeding up this process, in particular for target discovery. Indeed, similar strategies (i.e., profiling modified proteomes) have been successfully applied to identify phosphorylated substrates of bacterial kinase effectors such as *S. flexneri* OspG [28] and enteropathogenic *Escherichia coli* (EPEC) NleH [49]. Furthermore, understanding the reversal of ADP-riboxanation would provide valuable insights into the dynamic regulation of this modification. As the complexities of ADP-riboxanation are further unfolded, we can certainly expect a deeper understanding of the mechanisms underlying bacterial pathogenesis, which may open new avenues for therapeutic intervention during bacterial infections.

## Figures and Tables

**Table 1:** Post-translational modifications of host proteins catalyzed by bacterial effectors.

Bacteria	Effectors	Host Targets	PTMs	Functions	References
*Chromobacterium violaceum*	CteC	Ubiquitin	ADP-ribosylation	Blocks ubiquitin signaling	[6]
*Chromobacterium violaceum*	CopC	Caspase-3/7/8/9	ADP-riboxanation	Blocks cell death	[7,8]
Enteropathogenic *E. coli*	NleE	TAB2, TAB3	Cysteine methylation	Inhibits host NF-κB signaling	[9,10]
Enteropathogenic *E. coli*	NleB	FAS-associated death domain protein (FADD), TNFR1-associated death domain protein (TRADD)	GlcNAcylation	Inhibits death receptor-induced apoptosis	[11,12,13]
Enteropathogenic *E. coli*	Cif	Ubiquitin-like modifier NEDD8	Deamidation	Arrests cell cycle	[14]
*Legionella pneumophila*	SidEs	Rab1, Rtn4	Ubiquitination independent of E1/2	Interferes with vesicle trafficking and tubular ER	[15,16,17]
*Legionella pneumophila*	LnaB	Phosphoribosyl ubiquitin, Src	Phosphoryl-AMPylation	Activates NF-κB signaling, impairs phosphosignaling	[18,19]
*Legionella pneumophila*	MavC (Lpg2147)	Ubiquitin, UBE2N	Deamidation, transglutaminase-induced ubiquitination	Inhibits host NF-κB signaling	[20]
*Legionella pneumophila*	RavD	Linear ubiquitin chains	Deubiquitination	Inhibits linear ubiquitin chain-mediated signaling (e.g., NF-κB signaling)	[21]
*Legionella pneumophila*	RavZ	Atg8	Hydrolysis	Blocks autophagy	[22]
*Salmonella* Typhimurium	SopF	ATP6V0C	ADP-ribosylation	Blocks xenophagy	[23]
*Shigella flexneri*	OspI	UBC13	Deamidation	Inhibits host NF-κB signaling	[24]
*Shigella flexneri*	IpaH1.4, IpaH2.5	HOIL-1-interacting protein (HOIP)	Ubiquitination	Inhibits host NF-κB signaling	[25]
*Shigella flexneri*	OspCs	Caspase-4/11, eIF3	ADP-riboxanation	Blocks pyroptosis and protein translation, induces stress granules	[26,27]
*Shigella flexneri*	OspG	Cullin-associated NEDD8-dissociated protein 1 (CAND1)	Phosphorylation	Blocks septin cage assembly	[28]
*Shigella flexneri*	IpaH9.8	Guanylate-binding proteins (GBPs)	Ubiquitination	Inhibits GBP-mediated immunity	[29,30,31]
*Vibrio parahaemolyticus*	VopS	Rho, Rac, Cdc42	AMPylation	Inhibits actin assembly	[32]

## Data Availability

The original contributions presented in this study are included in the article. Further inquiries can be directed to the corresponding author.
